# Tryptophan Metabolism by Gut Microbiome and Gut-Brain-Axis: An *in silico* Analysis

**DOI:** 10.3389/fnins.2019.01365

**Published:** 2019-12-18

**Authors:** Harrisham Kaur, Chandrani Bose, Sharmila S. Mande

**Affiliations:** Life Sciences R&D, TCS Research, Tata Consultancy Services, Pune, India

**Keywords:** tryptophan metabolism, gut microbiome, gut-brain axis, genome mining, neurological disorders, *in silico*

## Abstract

The link between gut microbiome and brain is being slowly acknowledged due to the speculated role of resident gut microbial community in altering the functions of gut-brain axis (GBA). Recently, a number of microbial metabolites (referred to as neuro-active metabolites) produced through tryptophan metabolism have been suggested to influence the GBA. In view of this, the current study focuses on microbial tryptophan metabolism pathways which produce neuro-active metabolites. An *in silico* analysis was performed on bacterial genomes as well as publicly available gut microbiome data. The results provide a comprehensive catalog of the analyzed pathways across bacteria. The analysis indicates an enrichment of tryptophan metabolism pathways in five gut-associated phyla, namely, Actinobacteria, Firmicutes, Bacteroidetes, Proteobacteria, and Fusobacteria. Further, five genera, namely, *Clostridium*, *Burkholderia*, *Streptomyces*, *Pseudomonas*, and *Bacillus* have been predicted to be enriched in terms of number of the analyzed tryptophan metabolism pathways, suggesting a higher potential of these bacterial groups to metabolize tryptophan in gut. Analysis of available microbiome data corresponding to gut samples from patients of neurological diseases and healthy individuals suggests probable association of different sets of tryptophan metabolizing bacterial pathways with the etiology of different diseases. The insights obtained from the present study are expected to provide directions toward designing of microbiome based diagnostic and therapeutic approaches for neurological diseases/disorders.

## Introduction

Trillions of microbes residing on and within the human body (called microbiota) have been shown to play roles in health and disease (Shreiner et al., 2015; Wang et al., 2017). In particular, the microbes inhabiting the gastrointestinal tract (gut) aid in a number of metabolic functions, which include digestion and absorption of nutrients, regulation of gene expression, development of healthy immune system, and production of certain essential vitamins (Belkaid and Hand, 2014; Shreiner et al., 2015; Wang et al., 2017). The gut microbiota also helps in maintaining the integrity and the permeability of the intestinal epithelial cells, thereby protecting the host against invading pathogens and systemic inflammation (Kelly et al., 2015). On the other hand, disruption in the composition of gut microbiota has been found to be associated with the pathophysiology of several diseases and disorders like inflammatory bowel disease (IBD), colorectal cancer (CRC), diabetes, obesity, etc. (Becker et al., 2015; Baothman et al., 2016; Kaur et al., 2017; Dahmus et al., 2018). Intriguingly, apart from gut-associated metabolic diseases, an increasing body of evidences point toward association of gut microbiome with distant organs like brain, lung, etc. (Anand and Mande, 2018; Martin et al., 2018). For example, recent studies have speculated the link between dysbiotic gut microbiota and nervous system diseases/disorders like autism, multiple sclerosis, Parkinson’s disease, etc. (Kirby and Ochoa-Repáraz, 2018; Bedarf et al., 2019; Pulikkan et al., 2019). The focus of the current study pertains to the possible functional aspects of gut microbiome that influence the bi-directional communication between the gut and the brain, widely known as the “gut-brain axis” (GBA).

The GBA comprises of the central nervous system (CNS) including the brain and spinal cord, the autonomic nervous system, the enteric nervous system, and the hypothalmic-pituitary-adrenal (HPA) axis (Carabotti et al., 2015). The components of the GBA communicate and govern each other by means of various endocrine, immune, and neural pathways. The autonomous system is the central driver for two kinds of signals—(i) efferent signal which originates at enteric lumen and travels to the CNS and (ii) afferent signal which travels from the CNS to intestine (Carabotti et al., 2015). The HPA axis mediates the adaptive stress response of the host and primarily influences the release of stress hormone cortisol from the adrenal gland. This controlled release of cortisol in turn affects the emotional and cognitive function of the brain (Carabotti et al., 2015). Thus, the neural communication routes in combination with the hormonal pathways allow the brain to influence the function of the intestinal cells. Additionally, recent studies have suggested the effect of gut microbiota on the intestinal cells (Mayer et al., 2014; Carabotti et al., 2015). Thus, given the complex link between the gut microbiota and the host, the correlation of microbiome with the GBA is now being increasingly speculated.

Certain metabolites produced by gut microbiome have been shown to influence the GBA. For example, short chain fatty acids (SCFAs) like butyrate synthesized by gut microbiota are reported to stimulate memory and synaptic plasticity by inhibiting histone deacetylases (Vecsey et al., 2007; Stefanko et al., 2009). Butyrate has also been shown to influence the release of neurotransmitter serotonin from the intestinal enterochromaffin cells (Reigstad et al., 2015). Studies have also indicated that propionate produced by gut bacteria protects the blood–brain barrier (BBB) from oxidative stress (Hoyles et al., 2018). In addition, SCFAs can affect neuro-inflammation by modulating the production and recruitment of immune cells such as T cells, neutrophils, and inflammatory cytokines (Park et al., 2019).

Besides SCFAs, certain metabolites generated by gut bacteria can act as essential neuro-active molecules for the CNS. For example, few species of *Lactobacillus* and *Bifidobacterium* can produce neurotransmitters like acetylcholine and gamma-amino butyrate (GABA) (Cryan and Dinan, 2012; Galland, 2014). *Streptococcus*, *Enterococcus*, and *Escherichia* can synthesize serotonin, dopamine, and norepinephrine (Cryan and Dinan, 2012; Galland, 2014). However, the mechanisms through which the microbe-derived neurotransmitters evoke response in host are not well characterized. Additionally, some essential vitamins, such as vitamin K, B2, B9, and B12 synthesized by gut bacteria, can exert neuro-protective effects on the CNS (Parker et al., 2019).

The gut microbiota has been proposed to influence the GBA through various biochemical routes. Previous experimental reports have implicated several metabolites of bacterial tryptophan metabolism in modulating the health of the host (Roager and Licht, 2018). Furthermore, various studies have shown ability of certain bacteria like *Enterococcus, Pseudomonas*, etc., to produce one of the important neurotransmitters, serotonin, in tryptophan-rich media (Evrensel and Ceylan, 2015; Knecht et al., 2016). Studies have shown gut microbiota’s role in regulating the bioavailability of substrates that are required for biosynthesis of important neurotransmitters (Ney et al., 2017; Ma and Ma, 2019). For example, gut microbes can metabolize essential amino acid tryptophan as a precursor for synthesis of indole, serotonin, and melatonin, thereby limiting the availability of tryptophan for the host (O’Mahony et al., 2015; Martin et al., 2018). Additionally, bacterium like *Pseudomonas* has been shown to synthesize serotonin from the available tryptophan and utilizes the same for its virulence and inter-cellular signaling (Biaggini et al., 2015; Knecht et al., 2016). The reduction in the levels of circulating tryptophan by gut microbiota thus affects the serotonergic neurotransmission, thereby affecting the functioning of central and enteric nervous systems (O’Mahony et al., 2015; Jenkins et al., 2016). Low serotonin levels have been reported to have link with depression, fatigue, and impaired cognitive functions (Geldenhuys and Van der Schyf, 2011). Another example of tryptophan metabolism by gut bacteria pertains to production of tryptamine through the action of tryptophan decarboxylase (Williams et al., 2014). Tryptamine has been reported to influence cell’s inhibitory response to serotonin and release of serotonin by enterochromaffin cells (Zucchi et al., 2006; Williams et al., 2014). Other metabolites of tryptophan metabolism by gut bacteria, with proposed effects on brain and behavior, primarily include kynurenine, quinolinate, indole, and indole derivatives (O’Mahony et al., 2015; Gao et al., 2018). Kynurenine and quinolinate, for example, have been proposed to perturb brain functions and consequently cause depression like symptoms (Oxenkrug, 2013). Additionally, sequestering of host tryptophan to produce kynurenine and quinolinate have been suggested to reduce the concentration of tryptophan in blood, thus limiting the production of important neurotransmitters, such as serotonin in brain (Gao et al., 2018; Waclawiková and El Aidy, 2018b). Furthermore, indole and indole derivatives, including indole acetic acid (IAA) and indole propionic acid (IPA), have been shown to alter the metabolism of CNS in both human and animal studies (Furukawa et al., 2005; Puurunen et al., 2016; Jaglin et al., 2018). Overall, gut microbiome mediated tryptophan catabolism seems to be one of the crucial regulatory factors that is important for the GBA.

Given the significance of tryptophan metabolism in modulating the GBA, a comprehensive analysis of the corresponding pathways across bacteria is likely to improve our current understanding of microbiome-gut-brain axis. In the present study, the available bacterial genomes were computationally analyzed to understand the distribution of clinically significant (GBA associated) tryptophan metabolism pathways. Evaluation of gut microbiome’s role in brain diseases and disorders was also carried out using publicly available data. The insights obtained from the current study will be helpful in comprehending the understanding of host–microbiota interaction in context of gut-brain axis.

## Materials and Methods

### Selection of Neuro-Active Metabolite Production Pathways via Tryptophan Metabolism in Bacteria

One of the primary objectives of the present study pertains to the identification of bacterial tryptophan metabolizing pathways which leads to the production of metabolites that have been reported to influence the GBA. These metabolites include kynurenine, quinolinate, indole, IAA, IPA, and tryptamine (Agus et al., 2018; Roager and Licht, 2018). Further, earlier studies have reported bacterial pathways for production of serotonin and other indole derivatives like indole lactic acid, indole acrylic acid, and skatole through tryptophan metabolism (Galland, 2014; Gao et al., 2018). However, to the best of our knowledge, the enzymes involved in these pathways have not been experimentally characterized in bacteria. Thus, the current study does not include analysis of these pathways. Therefore, the final set of metabolites that was considered in the current study comprises of kynurenine, quinolinate, indole, IAA, IPA, and tryptamine. This set has been referred to as “TRYP-6” in the subsequent sections. An extensive literature survey was performed to collate information on experimentally characterized pathways/enzymes involved in the production of the “TRYP-6” metabolites for further computational analysis. The findings from literature mining are described in the following subsection.

### Literature Mining of Experimentally Characterized Tryptophan Metabolizing Pathways Associated With the Production of “TRYP-6”

Prediction of pathways is widely performed based on the presence of genes/proteins homologous to the experimentally identified genes/proteins involved in the particular pathway. However, this approach is limited in cases where one or more of the participating enzymes perform generic functions (example: hydrolases, oxidases, etc.) and thus are involved in multiple pathways. In order to address this limitation, the current study utilizes enzyme homology along with genomic proximity of the constituent genes for accurate prediction of a pathway. In addition, the pathways involving specific enzymes were also considered in the current analysis. Consequently, the present study includes *in silico* identification of the below mentioned three categories of pathways for production of “TRYP-6” in bacteria.

(i) Pathways experimentally characterized to have constituent genes in proximity on the genome;
(ii) Pathways comprised of enzymes specific to the corresponding reactions;
(iii) Pathways involving enzymes having specific functional domains.

**FIGURE 1:**
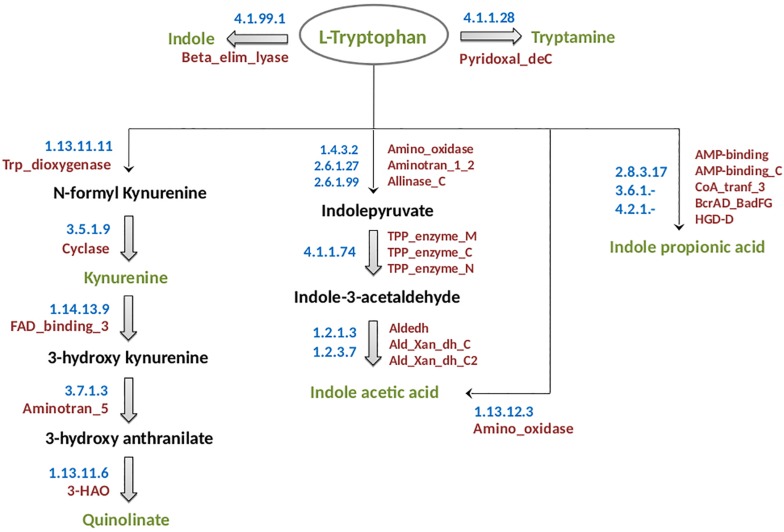
Schematic representation of tryptophan metabolism pathways leading to production of neuro-active compounds. The pathways correspond to metabolism of tryptophan (indicated in green font inside an oval) for production of six neuro-active compounds namely, indole, tryptamine, kynurenine, quinolinate, indole acetic acid, and indole propionic acid. These six compounds have been indicated in green font. The intermediate compounds of the pathways have been represented in black font. For each pathway, the EC numbers and the functional domains of the participating enzymes have been indicated in blue and red colors, respectively.

The experimentally characterized pathways for each of the analyzed neuro-active tryptophan metabolites of microbial origin are described below and have been schematically shown in Figure 1. Information on the enzymes involved in these pathways has been summarized in Supplementary Table S1. The methodologies followed for prediction of the “TRYP-6” production pathways are also described below.

(a) Kynurenine: The kynurenine pathway is a well-characterized pathway leading to production of nicotinamide adenine dinucleotide (NAD) in mammals (Nikiforov et al., 2015). The enzyme tryptophan 2,3 dioxygenase (TDO, EC 1.13.11.11) catalyzes the rate-limiting oxidation reaction of L-tryptophan to *N*-formyl-L-kynurenine (Kanai et al., 2009) (Figure 1). The genes encoding the two participating enzymes (including TDO) for kynurenine production, namely, TDO (*kynA*) and kynurenine formamidase (*kynB*) were reported to be present as a gene cluster in *Ralstonia metallidurans* (Kurnasov et al., 2003a). Further, in few other bacteria experimentally identified to produce kynurenine, such as, *Pseudomonas fluorescens*, the genes *kynA* and *kynB* are present in different locations in the corresponding genomes. It should be noted that the rate-limiting enzyme TDO comprises of a very specific functional domain, namely, Trp_dioxygenase (PF03301). Thus, in the current analysis, the pathway was predicted based on the presence of this functional domain across bacterial genomes.
(b) Quinolinate: Quinolinate, in addition to kynurenine, is also produced through the kynurenine pathway where kynurenine is further converted to quinolinate (Figure 1). The conversion of kynurenine to quinolinate has been experimentally reported to be catalyzed by three enzymes, namely, kynurenine 3-monooxygenase (KMO, EC 1.14.13.9), kynureninase (KYN, EC 3.7.1.3), and 3-hydroxyanthranilate-3,4-dioxygenase (HAD, EC 1.13.11.6) (Kurnasov et al., 2003a). The enzyme “HAD” contains a specific functional domain, namely, 3-HAO (PF06052.11). An analysis of genomic locations of the genes in quinolinate producing organisms (example: *Cytophaga hutchinsonii*, *P. fluorescens*, etc.) revealed that the genes were not always located as a cluster in the genomes. Hence, prediction of the pathway in bacteria was performed based on the presence of the specific functional domain 3-HAO as well as the other two domains FAD_binding_3 (PF01494.18) and Aminotran_5 (PF00266.18), corresponding to the enzymes KMO and KYN, respectively.
(c) Indole: The pathway for biosynthesis of indole from tryptophan is reported to be catalyzed by a single-step reaction involving a very specific enzyme tryptophanase (EC 4.1.99.1) (Snell, 1975). However, the functional domain (Beta_elim_lyase) of this enzyme was observed to be a part of numerous enzymes of varied metabolic pathways. Thus, “Blast” (Altschul et al., 1990) which considers the similarity of the whole protein sequence was performed to identify the homologs of tryptophanase. “Blast” was executed at an identity threshold of 70%, coverage threshold of 90%, and an e-value cut off of 1e-5. The query set consisted of the protein sequences of the homologs as obtained from “Uniprot” database^1^ and the target dataset contained the protein sequences of completely sequenced bacterial genomes obtained from NCBI^2^ and HMP^3^.
(d) IAA: IAA biosynthesis has been reported to be a characteristic of rhizosphere bacteria that regulate the growth of plants (Zhao, 2010). Additionally, IAA has been reported to be produced by bacteria inhabiting diverse habitats like aquatic ecosystems and gastrointestinal tract of animals (Patten et al., 2013). The production of IAA from tryptophan in plant bacteria *Pseudomonas syringae* has been shown to be facilitated by tryptophan-2-monooxygenase (EC: 1.13.12.3) (Gaffney et al., 1990). An alternate route for synthesis of IAA from tryptophan has been reported in the bacteria *Enterobacter cloacae* which are found in soil and gut (Schütz et al., 2003). The key enzyme of this pathway is indolepyruvate decarboxylase (EC 4.1.1.74) which catalyzes the conversion of indolepyruvate to indole-3-acetaldehyde. It is noteworthy that both the above-mentioned enzymes have specific activity for the corresponding reactions. Therefore, identification of bacteria having IAA production capability was performed by searching the homologs of these enzymes in bacterial genomes through “Blast” (Altschul et al., 1990) with an identity, coverage, and an e-value threshold of 70%, 90%, and 1e-5, respectively. The query set consisted of the protein sequences of the homologs retrieved from “Uniprot” database^4^ and the target comprised of the protein sequences of completely sequenced bacterial genomes from NCBI^6^.
(e) IPA: *In vivo* studies have reported that IPA can be produced by *Clostridium sporogenes* present in the gastrointestinal tract of humans (Wikoff et al., 2009). The conversion of tryptophan to IPA is catalyzed by a heterotrimeric phenyllactate dehydratase FldABC (Dodd et al., 2017). The reaction additionally requires a dehydratase activator (FldI) and an AMP-binding protein (FldL). The genes for the above-mentioned enzyme and proteins have been shown to be present in close proximity on the genome (Dodd et al., 2017). Hence, the prediction of IPA biosynthetic pathway was executed by utilizing the information of genomic locations of the participating genes on the genome, in addition to the presence of the corresponding protein homologs in a particular bacterium. Identification of protein homologs was performed based on the presence of the functional domains (retrieved from Pfam database; provided in Supplementary Table S1).
(f) Tryptamine: The pathway for the production of tryptamine has been suggested to involve a single-step decarboxylation reaction facilitated by the enzyme tryptophan decarboxylase (EC 4.1.1.28). It is noteworthy that this enzyme is a generic aromatic amino acid decarboxylase and is also involved in production of dopamine from tyrosine (Kuhar et al., 1999). Further, tryptamine which is a microbial derived monoamine has been reported to be similar to 5-hyroxytryptamine (clinically known as serotonin) in both structure and function (Bhattarai et al., 2018). Since tryptophan decarboxylase has been experimentally reported to be present in organisms, *C. sporogenes* and *Ruminococcus gnavus* (Williams et al., 2014), their homologs were searched in other bacteria. “Blast” (Altschul et al., 1990) was performed with parameters of identity, coverage, and e-value threshold of 70%, 90%, and 1e-5, respectively. The target dataset contained the protein sequences of completely sequenced bacterial genomes obtained from NCBI^4^ and HMP^5^.

Heat-map using “R” package “gplots”^7^ was generated to represent the proportion of strains of a gut-associated phyla predicted to harbor pathways for “TRYP-6” metabolites.

### Analysis of Tryptophan Transporters in Gut Bacteria

In addition to studying the enzymes involved in tryptophan metabolism, an attempt was made in the current study to analyze the transporters which import tryptophan from the surrounding environment into the bacterial cell. Such analysis would provide an idea about the capability of the bacteria (predicted to have the analyzed pathways) to sequester tryptophan from host. The most widely studied tryptophan specific transporter is TnaB, which is encoded by a gene operonic to the tryptophanase gene (*tnaA*; catalyzes the conversion of tryptophan to indole) in *Escherichia coli* (Gong and Yanofsky, 2003). Further, other non-specific transporters, such as AroP and Mtr, have been reported to be involved in tryptophan import (Li and Young, 2013). For example, the import of tryptophan is controlled only by AroP in *Corynebacterium glutamicum* (Wehrmann et al., 1995). Thus, in order to identify the pattern of distribution of tryptophan transporters across bacteria, the genomes of gut bacteria predicted to produce indole were screened for the presence of the three transporters, namely, TnaB, AroP, and Mtr. Since gene for TnaB is reported to be present close to *tnaA* gene (Gong and Yanofsky, 2003), the genomic location of the functional domain of TnaB was searched in proximity to the functional domain of tryptophanase enzyme. The presence of AroP and Mtr enzymes on gut bacterial genomes predicted to produce indole was characterized using “Blast” (Altschul et al., 1990). Blast was executed with identity, coverage, and e-value parameters of 70%, 90%, and 1e-5, respectively. The query protein sequences for the enzymes Mtr and AroP were retrieved from “Uniprot” database^8^. “Genevenn”^9^ was utilized to plot Venn diagram to depict distribution of transporters in gut bacteria. A heat-map was generated to represent the proportion of indole producing gut bacterial strains utilizing transporters. The heat-map was generated using “R” package “gplots”^7^ .

### Estimation of Tryptophan Metabolizing Potential of Bacteria

One of the primary focuses of the current study pertains to understanding any possible associations of the “TRYP-6” pathways with the gut microbiome of patients with neurological disorders. However, owing to the nature of the metagenomic datasets under study, identification of a bacterial strain harboring the analyzed pathways could be insufficient to establish any relevant inferences. Therefore, an evaluation of tryptophan metabolizing capability of bacteria at higher taxonomic levels (such as genera) was required. This evaluation was made for all bacterial groups analyzed at genera level. For each of the bacteria identified to have the selected tryptophan metabolizing pathway, a score “SCORBPEO” (Score for Bacterial Production of Neuro-active Compounds) was assigned. The “SCORBPEO,” for each pathway was determined by taking into account the proportion of bacterial strains predicted to have the pathway, the confidence value of the corresponding bacterial group, and a “gut-weightage” factor. The confidence value was included to allow a higher score for the bacterial taxa which had relatively higher amount of representative strains in the database. For the calculation of the confidence value, the number of strains present under a particular bacterial group was first noted. Further, based on this count, a percentile value was assigned and consequently a rank (between 1 and 10) was assigned to the corresponding bacterial group. Another factor called the “gut-weightage” was incorporated in “SCORBPEO” to assign a higher score to the bacterial genera having higher number of gut-associated strains with a predicted pathway. Therefore, a higher “gut-weightage” (for a genus) would indicate an enrichment of a pathway in the strains found in gut comparative to the strains found in other environments. Thus, the score “SCORBPEO” for a particular neuro-active compound “i” corresponding to a bacterial genus “j,” was computed using the following equation:

$$S\,C\,O\,R\,B\,P\,E\,O_{i\,j}=P*\alpha *\beta$$

where *P* represents the proportion of strains predicted with the compound “*i*” producing capability for the particular bacterial genus “*j*” (value of *P* ranges between “0” and “1”), α denotes the confidence value of the corresponding bacterial group (value of α ranges between “1” and “10”), and “β” corresponds to a “gut weightage” which represents an enrichment value of a particular pathway in the gut environment (value of β ranges between “1” and “5”). Thus, the values of the computed “SCORBPEO” scores ranged between 0 and 50. The values were further rescaled to “0–10.” For a particular pathway, a bacterial genus having a higher “SCORBPEO” would indicate a greater capability of production of a particular compound as opposed to a genus with a lower “SCORBPEO.”

Heat-map using “R” package “gplots”^10^ was generated to represent the normalized “SCORBPEO” scores of gut-associated genera predicted to harbor pathways for “TRYP-6” metabolites.

### Correlation of Tryptophan Metabolizing Potential With Gut Microbiome of Patients With Neurological Diseases/Disorders

In order to study any probable correlation of bacterial tryptophan metabolizing potential with the etiology of neurological diseases/disorders, gut microbiome data (16S rRNA) available from seven published studies were investigated. In particular, the gut microbiome data corresponding to three studies related to autism, two studies related to Parkinson’s disease, and one study on schizophrenia were analyzed. A summary on the analyzed datasets has been provided in Supplementary Table S2. The “sra” files for each of the datasets were extracted using SRA toolkit 2.3.4 (Leinonen et al., 2011). Subsequently, the obtained fastq sequences were quality filtered using “prinseq-lite” (Schmieder and Edwards, 2011). The sequences having an average phred score greater than or equal to 25 were retained for further analyses. For taxonomic classification of the sequences satisfying the quality threshold, naive Bayesian classifier of the “Ribosomal Database Project” (RDP classifier 2.10) (Wang et al., 2007) was utilized with a bootstrap confidence threshold of 80% for all taxonomic levels. In-house scripts were utilized to generate taxonomic abundances at phylum, class, order, family, and genus levels for each sample. The obtained taxonomic abundances were further normalized to generate relative abundances of taxa for every sample. The normalized taxonomic abundances at genera level were considered in the current study for further analysis.

In order to identify differentially abundant genera in healthy and diseased (autism, Parkinson’s disease, and schizophrenia) samples, “Wilcoxon rank-sum” test (Bauer, 1972) was performed on the datasets. R function “wilcox.test” was used to perform Wilcoxon rank-sum test. For each disease/healthy dataset pair (corresponding to seven studies), a list of differentially abundant genera was obtained with a *p*-value threshold of ≤ 0.01. Subsequently, a comparative analysis was performed between disease and healthy datasets with respect to—(a) abundances of bacterial genera predicted with the selected tryptophan metabolism pathways and (b) distribution of the pathways.

The association between differentially enriched genera and pathways for “TRYP-6” metabolites was represented using “Sankey” diagrams. Python library “plotly”^11^ was utilized to generate the same.

## Results

### Distribution of Tryptophan Metabolizing Pathways in Bacteria for Production of “TRYP-6” Compounds

**FIGURE 2:**
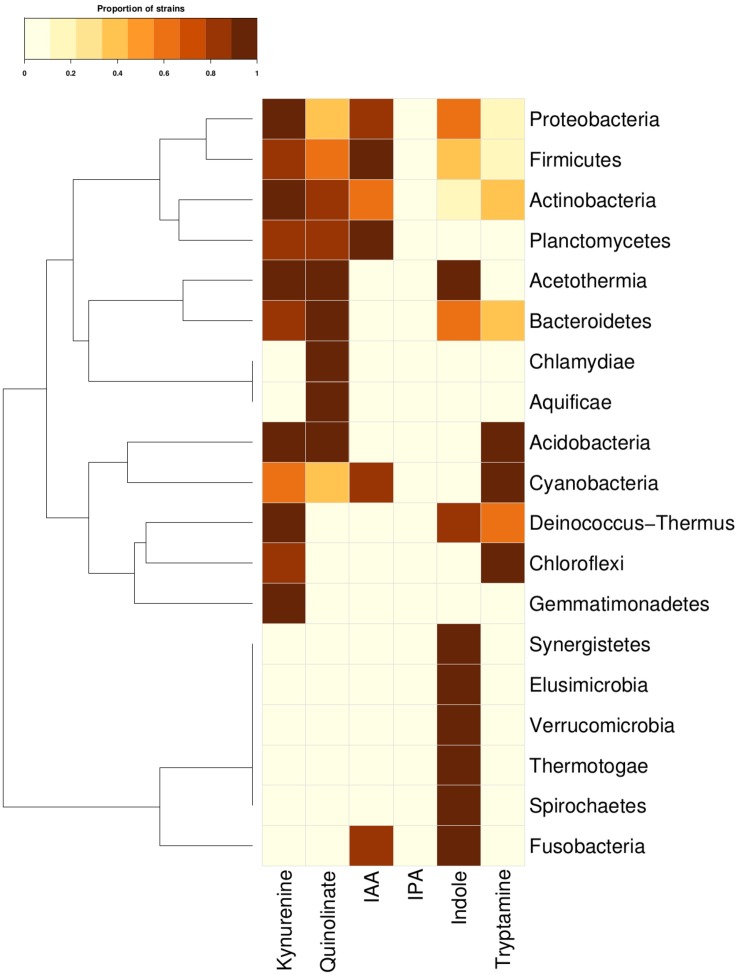
Tryptophan metabolism pathways for neuro-active compound production across bacterial phyla. Distribution of six neuro-active compound production pathways across bacterial phyla comprising of completely sequenced bacterial genomes. Each cell represents the proportion of strains predicted to harbor a pathway for a particular phylum.

In order to investigate the tryptophan metabolizing capability of bacteria in context of production of neuro-active compounds, genomes of 8392 completely sequenced bacteria (obtained from NCBI^12^ and HMP^13^) were analyzed using various computational approaches (details in section “Materials and Methods”). The results of the analyses indicated the presence of one or more tryptophan catabolism pathways in 19 phyla (Figure 2). These corresponded to approximately ∼40% (3389 out of 8392) of analyzed bacterial genomes (Supplementary Table S3). Three of the phyla, namely, Actinobacteria, Firmicutes, and Proteobacteria, known to commonly inhabit the gut, were found to have all six pathways for tryptophan utilization. Two other gut-associated phyla, namely, Bacteroidetes and Fusobacteria, were also observed to have tryptophan utilization capability, although using only four and two pathways, respectively. Association of Bacteroidetes and Proteobacteria with altered tryptophan metabolism in host has also been reported in literature (Wexler, 2007; Rizzatti et al., 2017). The current analyses therefore suggest a probable role of the five phyla, namely Actinobacteria, Firmicutes, Proteobacteria, Bacteroidetes, and Fusobacteria in affecting the tryptophan metabolism pathways in human gut.

The phyla-level analysis further provided a few interesting insights pertaining to specific tryptophan metabolism pathways. Four phyla (Bacteroidetes, Actinobacteria, Firmicutes, and Proteobacteria) were identified to be comparatively enriched in terms of proportion of tryptophan metabolizing strains (Figure 2). The results indicated Fusobacteria to be the highest enriched phyla in the indole production pathway with ∼72% of strains belonging to this phylum harboring the particular pathway (Figure 2). This observation corroborates with earlier reports that have experimentally shown production of indole through conversion of tryptophan in some of the species belonging to Fusobacteria (like *Fusobacterium nucleatum*) and role of this pathway in biofilm formation (Sasaki-Imamura et al., 2010). Three gut-associated phyla, namely, Bacteroidetes, Actinobacteria, and Proteobacteria, were relatively enriched for kynurenine pathway, with 26, 20, and 20% of strains possessing this particular pathway, respectively. This is in line with the previously reported observation of presence of this pathway in these three phyla (Lima et al., 2009). Interestingly, among these three phyla, the pathway for quinolinate production, which uses kynurenine as a precursor metabolite, was predicted as highly enriched (29%) in Bacteroidetes compared to that in Firmicutes (∼7%) and Proteobacteria (8%). This suggests that, in contrast to Firmicutes and Proteobacteria, most of the kynurenine producing strains under Bacteroidetes are capable of further converting it to quinolinate. Overall, the results indicate probable potential of bacterial groups that are common inhabitants of human gut, to utilize tryptophan for production of metabolites like kynurenine, quinolinate, tryptamine, indole, and indole derivatives (IAA and IPA). The depletion of the available tryptophan in turn may affect human metabolism.

**FIGURE 3:**
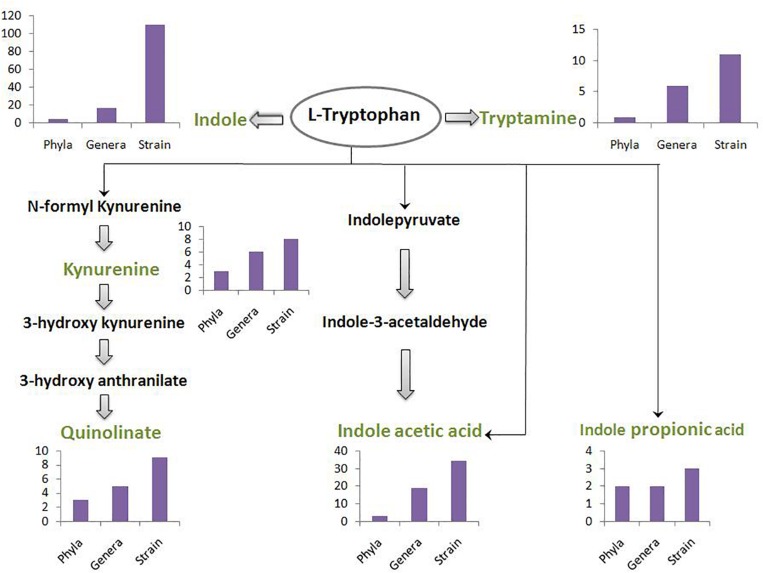
The count of gut bacteria at phyla, genera, and strain levels predicted to possess each of the six analyzed pathways. The pathways pertain to metabolism of tryptophan (indicated in black font inside an oval) for production of six neuro-active compounds namely, indole, tryptamine, kynurenine, quinolinate, indole acetic acid, and indole propionic acid. These six compounds have been indicated in green font. The intermediate compounds of the pathways have been represented in black font. The bar plot depicting phyla-count, genera-count and strain-count for each pathway has been placed behind or below the corresponding neuro-active compound.

A schematic diagram has been depicted in Figure 3 which shows the number of gut-associated phyla, genera, and strains predicted to possess the analyzed pathways. Figure 3 provides an overall idea about enrichment of the analyzed pathways in gut bacteria. As seen in Figure 3, the pathway for indole production was predicted to be most enriched with phyla count 5, genera count 17, and strain count 110. The enrichment of indole production pathway was followed by the pathways for IAA, tryptamine, quinolinate, kynurenine, and IPA, respectively. While the IAA production pathway was predicted in 34 gut bacterial strains, the strain count for tryptamine, quinolinate, and kynurenine production pathways was observed to be ∼10. Further, IPA production pathway was obtained as the lowest enriched pathway with strain count as only 3. Overall, the result suggests relatively higher capability of tryptophan metabolism through the routes producing indole and IAA (among “TRYP-6”) by gut bacteria.

### Tryptophan Metabolizing Capability of Gut Bacteria for Production of “TRYP-6” Compounds

**FIGURE 4:**
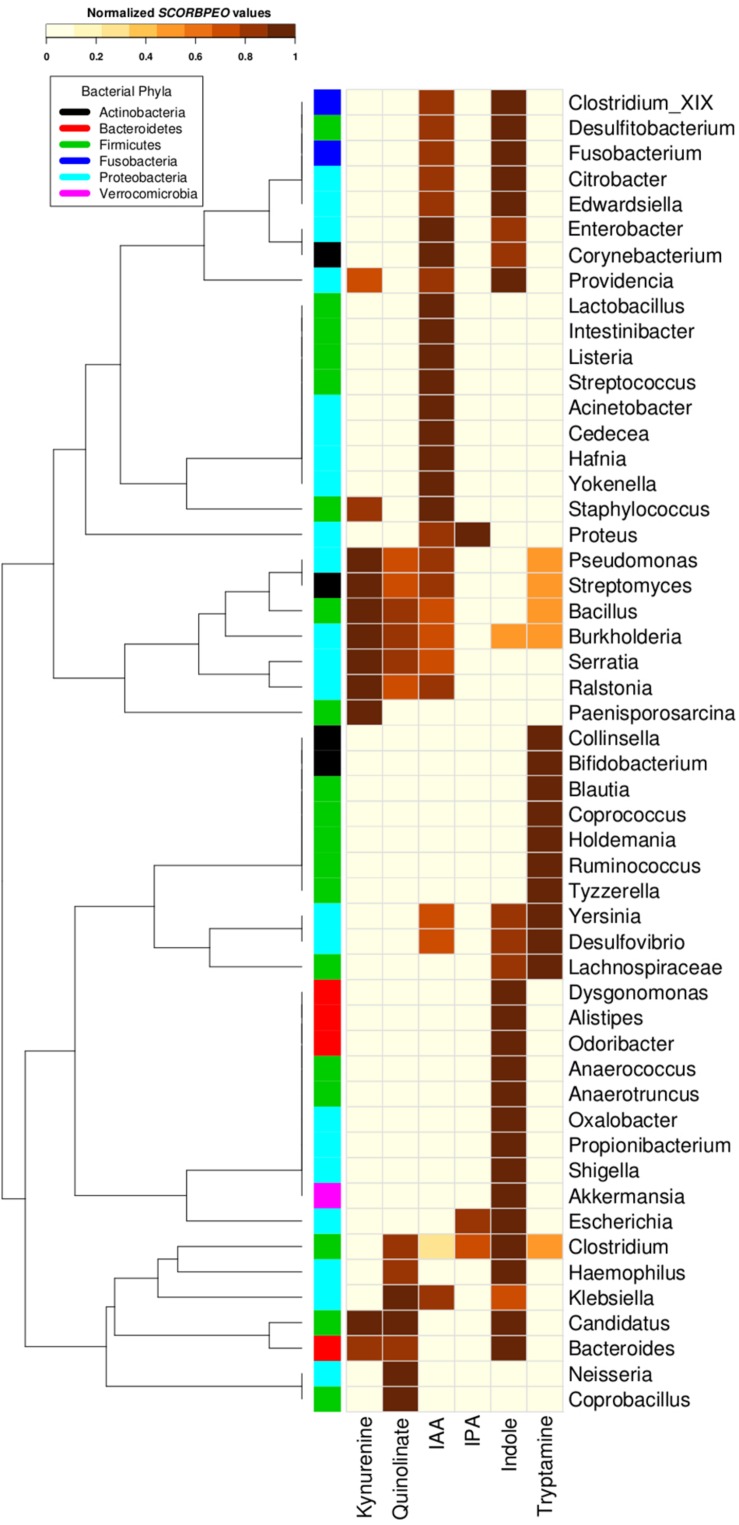
Tryptophan metabolism pathways for neuro-active compound production in bacterial genera found in gut. Distribution of six neuro-active compound production pathways across genera comprising of completely sequenced bacterial genomes found in human gut. Each cell represents “SCORBPEO” (Score for Bacterial Production of Neuro-active Compounds) value corresponding to the pathways in these genera. “SCORBPEO” indicates the relative capabilities for production of a particular neuro-active compound of any genus, evaluated based on the number of pathway containing strains, the database size of the respective genus, and enrichment of the pathway in gut-associated strains of the genus.

Going deeper at genera level, a clear occurrence of high proportions of tryptophan utilizing bacterial strains in gut environment was observed. In order to understand potential of utilization of tryptophan by various genera, each genus belonging to the gut-associated phyla (Bacteroidetes, Proteobacteria, Firmicutes, Actinobacteria, and Fusobacteria) having tryptophan metabolizing capability was assigned a score “SCORBPEO” (described in section “Materials and Methods”). A higher “SCORBPEO” value for a given bacterial genus would indicate a greater probability of production of the particular metabolite in comparison to a genus with lower “SCORBPEO” value. An in-depth investigation of the selected pathways in the bacterial genera previously reported to be associated with gut indicated 52 gut genera (out of total 93 gut genera) to have at least one of the six analyzed pathways (Supplementary Table S4). For each of these 52 genera, the “SCORBPEO” values across the pathways under study have been shown in Figure 4. The results suggest differences in pathway distribution in evolutionarily close gut genera belonging to a particular phylum. One of the reasons could be that these bacterial genera might have acquired the analyzed pathways to adapt to the environment they inhabit.

The pathway distribution profile (Figure 4) indicated that gut genera *Clostridium* and *Burkholderia* contained five out of the six analyzed pathways for production of neuro-active metabolites. While kynurenine production pathway was found to be absent in *Clostridium*, *Burkholderia* did not possess required genes for IPA production. Further, out of six analyzed pathways, *Streptomyces*, *Pseudomonas*, and *Bacillus* genus were observed to have only four pathways (for production of kynurenine, quinolinate, IAA, and tryptamine). Also, the observed high “SCORBPEO” value (10) of *Pseudomonas* for kynurenine pathway indicates a higher probability of tryptophan metabolism via this pathway in the gut environment colonized by *Pseudomonas* strains. This is in line with the experimentally characterized enzymes of this pathway in *Pseudomonas aeruginosa* (Bortolotti et al., 2016). These results suggest probable role of the bacterial genera *Clostridium*, *Burkholderia*, *Pseudomonas*, *Streptomyces*, and *Bacillus* in utilizing tryptophan for production of neuro-active compounds in the gut, thereby reducing the availability of tryptophan for the host.

The pathway distribution profile indicated that, among the six analyzed pathways, IAA and indole production pathways occurred in highest number of gut genera (28 and 27, respectively) (Figure 4). *Bacillus*, *Klebsiella*, *Ralstonia*, and *Staphylococcus* were found to have relatively higher capability of producing IAA. IAA, other than being implicated in neurological disorders, has widely been studied for its role as aryl hydrocarbon receptor (AhR) in regulation of intestinal immunity (Gao et al., 2018). IAA has also been proposed to have a significant role in bacterial signaling and colonization (Spaepen et al., 2007). Several gut bacteria including *Clostridia*, *Bacteroides*, and *Escherichia* have been characterized by earlier studies to produce IAA through tryptophan metabolism (Gao et al., 2018). The predicted indole producing genera having a greater “SCORBPEO” (≥ = 2) value were observed to be *Bacteroides*, *Citrobacter*, *Clostridium_XIX*, *Desulfitobacterium*, *Edwardsiella*, *Escherichia*, *Fusobacterium*, *Providencia*, and *Shigella* (Figure 4). Previous reports have indicated that a wide array of Gram-positive as well as Gram-negative bacteria produce indole from the available tryptophan and utilize the same as a major intercellular signaling molecule (Jaglin et al., 2018). Apart from this, indole produced by bacteria has been shown to be involved in bacterial sporogenesis, biofilm formation, drug resistance, virulence, and disease pathogenesis in host (Hu et al., 2010; Lee and Lee, 2010; Gagnière et al., 2016). Thus, the results of current analysis suggest probable role of indole and IAA produced by *Bacillus*, *Klebsiella*, *Ralstonia*, *Staphylococcus*, *Bacteroides*, *Citrobacter*, *Clostridium_XIX*, *Desulfitobacterium*, *Edwardsiella*, *Escherichia*, *Fusobacterium*, *and Providencia* in colonization of normal flora in the gut. In addition, these bacteria-derived compounds (IAA, indole) may also contribute to virulence through facilitating pathogenic colonization in cases of bacterial infection.

Among the remaining four tryptophan metabolizing pathways (under study), those involved in production of tryptamine, kynurenine, and quinolinate were predicted in 15, 11, and 10 gut genera, respectively. While *Pseudomonas*, *Bacillus*, *Burkholderia*, and *Streptomyces* had higher “SCORBPEO” values (>5) for kynurenine production pathway, *Klebsiella*, *Bacillus*, and *Burkholderia* were predicted to have higher “SCORBPEO” value (≥2) for quinolinate production pathway (Figure 4). Earlier experimental studies have identified these pathways in few species belonging to *Pseudomonas*, *Burkholderia*, *Streptomyces*, *Ralstonia*, *Cyanidium*, *Cytophaga*, *Karlingia*, and *Xanthomonas* (Kurnasov et al., 2003b; Colabroy and Begley, 2005). Kynurenine has also been implicated in bacterial signaling and colonization, similar to that by indole and indole derivatives (Farrow and Pesci, 2007). On the other hand, quinolinate has been shown to be involved in pathogenesis of neurological diseases (Lugo-Huitrón et al., 2013). Thus, abundance of *Bacillus*, *Burkholderia*, *Pseudomonas*, and *Streptomyces* (predicted to have higher “SCORBPEO” values for kynurenine pathway) may indicate involvement of kynurenine as a major (bacterial) inter-cellular signaling molecule in gut.

The genera predicted to harbor tryptamine production pathway with comparatively higher “SCORBPEO” values (> 0.5) included *Holdemania*, *Tyzzerella*, *Desulfovibrio*, and *Yersinia* (Figure 4). *Bacillus*, *Clostridium*, and *Ruminococcus* genera were also found to have this pathway, although with lower “SCORBPEO” values. This pathway has been experimentally characterized by earlier studies in *Bacillus atrophaeus*, *C. sporogenes*, and *R. gnavus* (Williams et al., 2014). The current results, along with previous literature, therefore suggest probable role of certain genera, namely, *Holdemania*, *Tyzzerella*, *Desulfovibrio*, *Yersinia*, *Bacillus*, *Clostridium*, and *Ruminococcus* in regulating serotonin release through tryptamine production, thereby positively influencing host neurophysiology, in contrast to the negative effect of quinolinate as discussed above.

The last tryptophan metabolizing pathway (under study), namely, production of IPA from tryptophan, was observed to be present in only three gut genera (*Clostridium*, *Escherichia*, and *Proteus*), indicating that synthesis of IPA in the gut is performed by limited gut bacterial groups. IPA production by certain species of *Clostridium* (such as *C. sporogenes*) in the gut has also been reported in literature (Jellet et al., 1980; Wikoff et al., 2009; Dodd et al., 2017). Like tryptamine, IPA is also known to be a neuro-protectant which works as an anti-oxidant against a variety of oxidotoxins (Chyan et al., 1999; Bendheim et al., 2002). Thus, the presence of IPA producing strains of *Clostridium*, *Escherichia*, and *Proteus* genera in gut may confer protective effect on brain function.

### Profiling of Tryptophan Transporters in Gut Bacterial Genomes

In order to identify organisms that have not only the potential to metabolize tryptophan, but also have the capability to sequester tryptophan from the host, predicted gut bacterial genomes having tryptophan metabolizing pathways were screened further for the presence of tryptophan specific transporters. TnaB is the most widely studied tryptophan specific transporter. It is encoded by a gene operonic to the tryptophanase gene (tnaA). The gene tnaA catalyzes the conversion of tryptophan to indole (Gong and Yanofsky, 2003; Li and Young, 2013). In addition, other non-specific transporters, such as AroP and Mtr, have also been reported to be involved in tryptophan import (Wehrmann et al., 1995; Li and Young, 2013).

**FIGURE 5:**
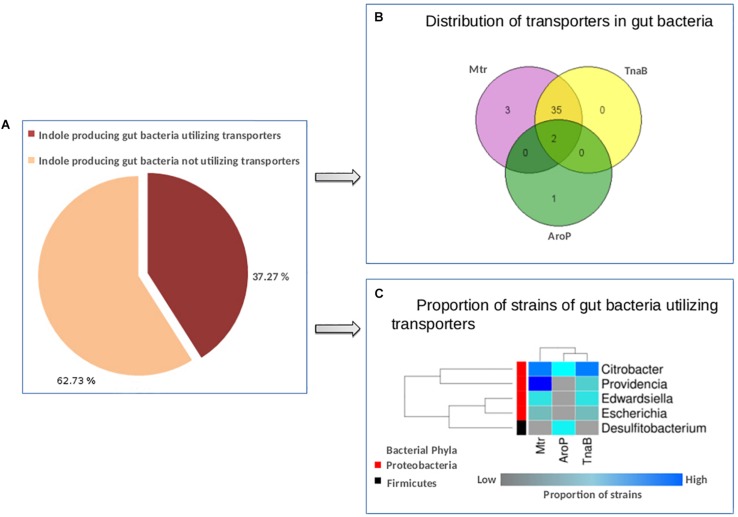
Distribution of tryptophan transporters in gut bacteria. **(A)** Pie-chart depicting the proportion of indole producing gut bacteria predicted to have tryptophan transporters versus those not utilizing the same. **(B)** Venn diagram representing the distribution profile of three known transporters namely, TnaB, Mtr, and AroP in gut bacteria. **(C)** Heat-map showing the proportion of strains in each of the five gut bacterial genera predicted to utilize the three tryptophan transporters (TnaB, Mtr, and AroP).

The results suggest that out of 110 gut bacteria predicted to have tryptophan metabolism pathways (in context to indole production, as discussed in section “Materials and Methods”), 41 (37.2%) organisms utilize one or more tryptophan transporters for sequestering tryptophan from the host (Figure 5A). A list of bacterial strains predicted to have any of the transporters has been provided in Supplementary Table S5. Interestingly, 85% of indole producers were predicted to utilize the two tryptophan transporters, i.e., Mtr and TnaB (Figure 5B). These included strains of four genera namely, *Citrobacter*, *Escherichia*, *Edwardsiella*, and *Providenci*a, all belonging to phylum Proteobacteria. On the other hand, only three strains (*Citrobacter freundii*_4_7_47CFAA, *Citrobacter* sp._30_2, and *Desulfitobacterium hafniense*_DP7) were predicted to utilize the low affinity/non-specific AroP transporter (Figure 5B). While the genus *Citrobacter* belongs to Proteobacteria phylum, *Desulfitobacterium* belongs to the phylum Firmicutes. Thus, while Mtr and TnaB are probably specific to Proteobacteria, AroP is a more generic one shared by other phyla. A heat-map showing the proportion of strains in the five genera predicted to have tryptophan transporters has been depicted in Figure 5C. Furthermore, the two strains of *Citrobacter*, namely, *Citrobacter freundii*_4_7_47CFAA and *Citrobacter* sp._30_2 were observed to harbor all the three studied tryptophan transporters (Supplementary Table S5 and Figure 5C), suggesting that these bacterial strains are probably more robust in acquisition of tryptophan from host under varying levels of this amino acid. Further, some organisms predicted to produce indole, such as *Alistipes*, *Akkermansia*, *Burkholderia*, *Clostridium*, etc., were observed to lack any of the three investigated transporters, suggesting the presence of hitherto uncharacterized non-specific amino acid transporters in these organisms.

**FIGURE 6:**
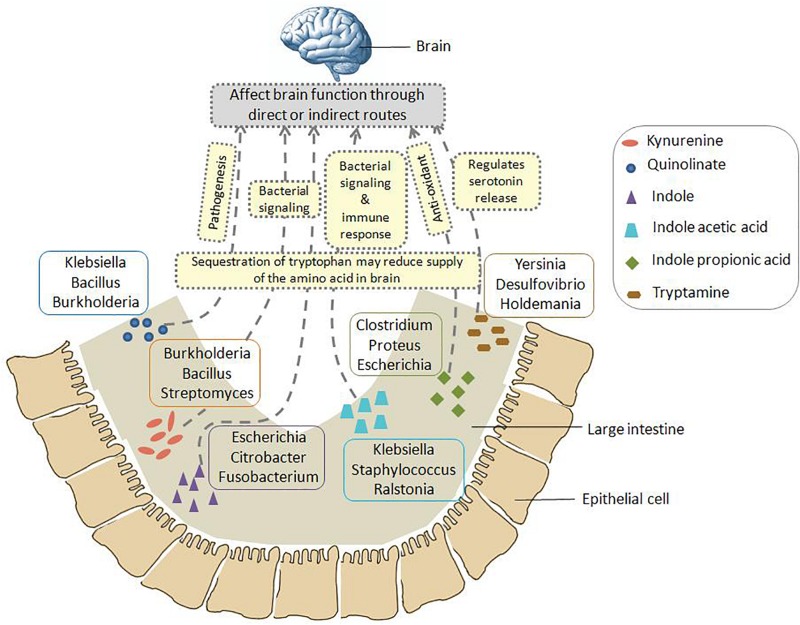
Schematic representation of the insights obtained from genomic analysis in context of tryptophan metabolism pathways in gut bacteria and their probable connection to brain. The six neuro-active compounds produced through bacterial tryptophan metabolism are depicted using various shapes as shown in the legend provided inside the figure. For each compound, the top three genera with respect to the “SCORBPEO” value are shown within rectangles having solid borders. For example, bacterial genera such as *Klebsiella*, *Staphylococcus*, *Ralstonia*, etc., produce indole acetic acid (IAA) in the large intestine through tryptophan metabolism. Probable ways by which each of the six compounds may alter the functioning of GBA (as collated from literature) have been indicated by yellow rectangles having dotted border. For instance, IAA may affect the interaction between gut and brain by acting as inter-cellular signaling molecule and immune system modulator. Apart from such mechanisms mediated by tryptophan metabolism products, sequestration of tryptophan by gut bacteria may have a direct impact on brain tryptophan level, which in turn can affect brain function.

A schematic representation has been shown in Figure 6 which summarizes the insights obtained from various genomic analysis performed in the current study (as described above). Figure 6 provides information on the probable bacterial groups that are involved in tryptophan metabolism in gut, thus producing metabolites such as kynurenine, quinolinate, indole, IAA, IPA, tryptamine, etc. These metabolites may have a role in altering the functioning of GBA through various direct and indirect processes. Such processes involve pathogenesis, bacterial signaling, production of anti-oxidants, immune system modulation, regulation of serotonin release, etc. Apart from such mechanisms, sequestration of tryptophan by the bacterial groups depicted in Figure 6 may affect the brain function as the tryptophan level reduces in brain. Overall, the information portrayed in Figure 6 would help in designing experiments toward better understanding the crosstalk between gut microbiome and brain.

### Link Between Tryptophan Metabolizing Potential of Gut Microbiome and Neurological Disorders

Since studies have indicated link between tryptophan metabolism by gut microbiome and neurological disorders like autism, Parkinson’s disease, depression, schizophrenia, etc. (Kałużna-Czaplińska et al., 2017; Agus et al., 2018; Waclawiková and El Aidy, 2018a), available gut microbiome (16S rRNA) datasets corresponding to patients suffering from three neurological disorders were analyzed. Details on the sample size corresponding to the analyzed datasets are provided in Supplementary Table S2. In order to capture variations in tryptophan metabolizing potential of gut microbiomes in each of the studied neurological disorder, differentially abundant gut genera between diseased and matched healthy cohorts were analyzed. The results of the analysis pertaining to three analyzed disorders are described in the following subsections.

#### Tryptophan Metabolism by Gut Bacteria in Autism

**FIGURE 7:**
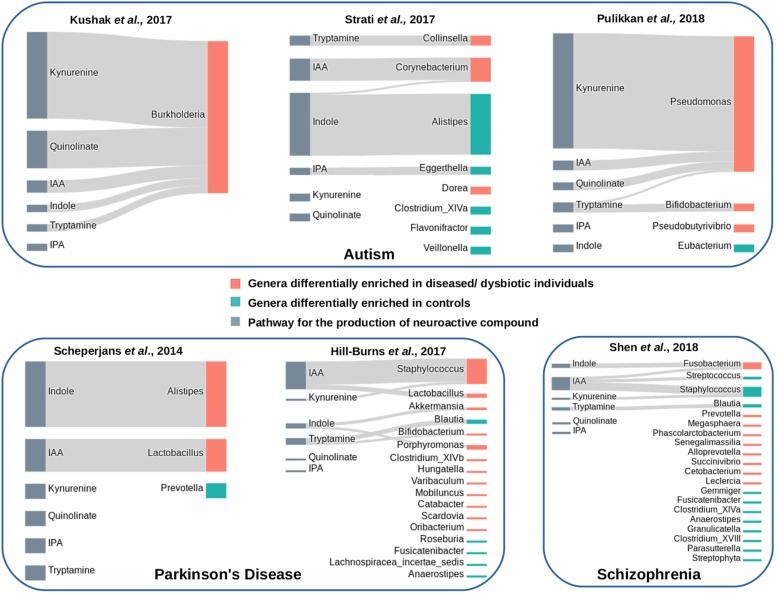
Tryptophan metabolism pathways in differentially abundant genera in gut microbiome of patients suffering from neurological diseases and healthy individuals. Each panel corresponds to a set of microbiome data obtained from patients of a neurological disease and matched healthy individuals. The genera differentially enriched in diseased cohorts have been shown with orange bars, while those differentially abundant in healthy cohorts have been shown with cyan bars. The pathways that have been predicted in differentially abundant genera in diseased or healthy individuals have been represented by Sankey diagrams. The width of the pathway-genus association represents the “SCORBPEO” value of the genus for the respective pathway.

Three publicly available gut microbiome datasets corresponding to autistic and matched healthy children were analyzed (Supplementary Table S2). These included one dataset obtained from duodenal mucosa (Kushak et al., 2017) and two datasets obtained from fecal samples (Strati et al., 2017; Pulikkan et al., 2018). The obtained results indicated enrichment of tryptophan metabolism pathways in autism (Figure 7). For the mucosa samples, *Burkholderia* was found to be significantly abundant genus (with *p*-value *leq* = 0.01) with five out of the six analyzed tryptophan pathways in autistic children (Figure 7). A higher “SCORBPEO” score (described in section “Materials and Methods”) of 8.61 corresponding to the kynurenine pathway suggests a higher utilization of tryptophan through the kynurenine pathway by *Burkholderia* in the gut of autistic children. *Burkholderia* was also predicted to harbor quinolinate pathway with a “SCORBPEO” score of 3.75. Further, the genera *Pseudomonas*, harboring kynureine, quinolinate, and IAA production pathways, were observed to be relatively abundant in one of the analyzed fecal datasets (Figure 7). *Pseudomonas* was found to have highest “SCORBPEO” score of 10 for kynurenine production. Considering that earlier studies have also indicated secretion of clinically significant level of kynurenine in *P. aeruginosa* (Bortolotti et al., 2016), *Pseudomonas* probably plays a key role in contributing in the disease etiology by affecting tryptophan metabolism. Earlier reports have also suggested kynurenine and quinolinate as neurotoxic metabolites, which are involved in disrupting neurotransmission, thereby leading to depression and altered brain function (Schwarcz et al., 2012; Waclawiková and El Aidy, 2018a). Kynurenine, in addition, has also been reported to have the ability to cross the BBB (Fukui et al., 1991; Catena-Dell’Osso et al., 2013). Thus, kynurenine, if produced in excess amount in gut, probably enters the blood circulation and eventually invade BBB.

Apart from kynurenine and quinolinate production pathways, present analyses suggest enrichment of pathway for production of indole and the indole derivative IAA by *Burkholderia*, *Pseudomonas*, and *Corynebacterium*in autistic children (Figure 7). Since earlier studies have suggested involvement of indole and IAA in bacterial signaling and colonization (Spaepen et al., 2007; Sasaki-Imamura et al., 2010), it is likely that indole and IAA producing strains of the genera *Burkholderia*, *Pseudomonas*, and *Corynebacterium*in probably help in pathogen colonization in the gut which negatively influence the functioning of GBA in autism. In contrast to that observed in autistic datasets, very few relatively abundant genera in the microbiome of healthy (control) children were found to possess the analyzed tryptophan metabolizing pathways (Figure 7). These included an indole producing genus *Alistipes* and an IAA producing genus *Eggerthella* with lower “SCORBPEO” values 1.5 and 0.2, respectively. Thus, it is likely that these genera utilize the secreted indole and IAA in inter-bacterial signaling, which in turn contributes to maintenance of a “healthy” gut microbiome.

#### Tryptophan Metabolism by Gut Bacteria in Parkinson’s Disease

Analysis of Parkinson’s disease microbiome included two publicly available datasets (Scheperjans et al., 2015; Hill-Burns et al., 2017) corresponding to the disease and matched healthy controls (Supplementary Table S2). The present analysis indicated an enrichment of indole production pathway in diseased condition (Figure 7). The indole producing genera which were relatively abundant in disease (in any one of the datasets under study) included *Akkermansia*, *Alistipes*, and *Porphyromonas*. Apart from indole, two genera predicted with IAA production pathway (*Lactobacillus* and *Staphylococcus*) were seen to be differentially abundant in diseased condition (Figure 7). One of these genera, namely, *Lactobacillus*, was obtained as differentially abundant in two of the analyzed datasets. One of the strains of *Lactobacillus*, namely, *Lactobacillus reuteri*, has been reported to affect the functioning of enteric nervous system by modulating gut motility and perception of pain (Kunze et al., 2009). Thus, the obtained results in the present study probably indicate likely involvement of the above-mentioned bacterial groups (*Akkermansia*, *Alistipes*, *Porphyromonas*, *Lactobacillus*, and *Staphylococcus*) in the disease etiology through production of indole and IAA, and thereby, altering tryptophan homeostasis in gut.

Further, unlike in autism, the analysis indicated kynurenine and quinolinate pathways not to be distinctive in the analyzed datasets of Parkinson’s disease. *Staphylococcus*, obtained as differentially abundant in one of the analyzed Parkinson’s dataset, was predicted to have low probability of utilization of bacterial kynurenine and quinolinate pathways (based on lower “SCORBPEO” value of 0.09). Thus, the present analysis highlights significance of bacterial utilization of tryptophan through different sets of metabolic routes in different neurological disorders.

#### Tryptophan Metabolism by Gut Bacteria in Schizophrenia

Analysis of gut microbiome corresponding to Schizophrenia included the only publicly available dataset by Shen et al. (2018). Unlike in the previous two disorders (autism and Parkinson’s), the distinction between diseased and healthy datasets based on the analyzed pathways was not very clear in schizophrenia. *Fusobacterium*, one of the differentially abundant genera in Schizophrenia, was predicted to have indole production pathway with a relatively higher “SCORBPEO” value of 4.42 (Figure 7). *Fusobacterium* also had the predicted pathway for IAA production, however, with a lower “SCORBPEO” value of 0.14, thus indicating very low probability of IAA production by this genus. The healthy dataset was also observed to be relatively enriched in the genus *Staphylococcus* (among others), having IAA production pathway with a higher “SCORBPEO” value of 7.48. Thus, correlation of the pathways under study with the disease etiology of schizophrenia, if any, is not very clear from the current analysis. Only the pathway for tryptamine production which was not enriched in disease microbiome was predicted in a relatively abundant genus *Blautia* in healthy microbiome (Figure 7). Tryptamine has been reported to influence cell’s inhibitory response to serotonin and release of serotonin by enterochromaffin cells (Zucchi et al., 2006; Williams et al., 2014). Availability of more microbiome data related to Schizophrenia will be helpful in deciphering probable implication of the analyzed bacterial pathways in this neurological disorder.

## Discussion

The present study provides a comprehensive catalog (based on *in silico* analyses) of bacterial tryptophan metabolism pathways for production of the neuro-active “TRYP-6” compounds (kynurenine, quinolinate, indole, IAA, IPA, and tryptamine). Results of the analysis revealed a relative enrichment of these pathways in bacterial phyla commonly found in the gut, such as Actinobacteria, Bacteroides, Firmicutes, Proteobacteria, and Fusobacteria, suggesting probable role of these bacteria in the functioning of GBA. Further, the “SCORBPEO” values assigned to each of the gut-associated bacterial genus provided an estimate of these bacteria’s potential for production of “TRYP-6” compounds. Such estimate is specifically helpful in inferring functional potential of a given microbiome (inhabiting a particular niche like human gut) in cases where taxonomic composition can be resolved only up to genera level due to technological limitations. Analysis of the “SCORBPEO” score suggested higher potential of tryptophan metabolism for some of the genera (such as *Burkholderia*, *Pseudomonas*, *Ralstonia*, *Klebsiella*, *Citrobacter*, etc.) belonging to the phylum Proteobacteria. Increase in the abundance of Proteobacteria has also been reported to be correlated with brain diseases and disorders (Branton et al., 2013; Rogers et al., 2016; Rizzatti et al., 2017). It is likely that the above-mentioned gut genera belonging to phylum Proteobacteria utilize tryptophan from host and metabolize it to produce neuro-active compounds, thus modulating the function of GBA and contributing to the etiology of neurological diseases and disorders (as depicted in Figure 6).

One of the interesting outcomes of the current analysis pertains to prediction of five out of the six analyzed pathways in the gut genera *Clostridium* and *Burkholderia*. Production of indole, indole derivatives, and tryptamine through tryptophan metabolism has been characterized earlier in *Clostridium* (Williams et al., 2014; Gao et al., 2018). Our results, in addition, indicated potential of bacterial strains belonging to this genus to produce quinolinate. Among the gut-associated strains, *Clostridium hathewayi* WAL 18680 was predicted to have the quinolinate pathway. However, the genes involved in production of the precursor (of quinolinate pathway) kynurenine were absent in this strain. This probably suggest a shared metabolism for quinolinate pathway where a group of organisms like *Clostridium* utilize kynurenine as a substrate which is produced (and also utilized) by a second group of bacteria like *Bacillus*, *Burkholderia*, *Pseudomonas*, etc. Further, the current results suggest probable presence of all the analyzed pathways except the one for the production of IPA in *Burkholderia*. Previous studies have reported production of kynurenine and quinolinate in various strains of *Burkholderia* (Kurnasov et al., 2003b; Colabroy and Begley, 2005). Apart from these two pathways, our analysis predicted the pathways for production of indole, IAA, and tryptamine in *Burkholderia*, although with lower “SCORBPEO” values. Therefore, the current results suggest a relatively higher potential of *Clostridium* and *Burkholderia* to affect host tryptophan metabolism in the gut.

Analysis of microbiome data of different neurological disorders suggested probable involvement of different sets of bacterial tryptophan metabolism pathways in the disease etiology. For instance, the results indicated an enrichment of kynurenine and quinolinate pathways, mostly driven by bacterial groups like *Burkholderia* and *Pseudomonas* in autism. On the other hand, the current analysis suggested probable effect of tryptophan utilization via the pathways for production of indole and indole derivatives, by the bacterial genera like *Alistipes* and *Staphylococcus* in the etiology of Parkinson’s disease. It is noteworthy that some of the metabolites under study, such as indole, IAA, and kynurenine, also function as intra-bacterial signaling molecules. Thus, further experimental investigations would be required to decipher the role of the predicted tryptophan metabolism pathways in *Burkholderia*, *Pseudomonas*, *Alistipes*, and *Staphylococcus*.

$$S\,C\,O\,R\,B\,P\,E\,O_{i\,j}=P*\alpha *\beta .$$

## Conclusion

In the present work, we have attempted to understand the communication between gut bacteria, tryptophan metabolism, and neurological pathologies. A comprehensive *in silico* analysis on the available bacterial genomes and gut microbiome data of patients suffering from neurological disorders was performed. Our analyses provide an exhaustive catalog of the six analyzed tryptophan metabolism pathways (leading to production of kynurenine, quinolinate, indole, IAA, IPA, and tryptamine) across bacterial groups. This knowledgebase is expected to be useful resource for future studies on GBA. The results further indicate a relative enrichment of tryptophan metabolizing capabilities in bacterial groups commonly associated with gut environment. The analyses of microbiome data indicate association of distinct modules of bacterial tryptophan metabolism with the etiology of different neurological diseases such as, autism, Parkinson’s, and schizophrenia. These insights are expected to aid future experiments which can enhance our understanding of microbiome-GBA and may also help in designing microbe-based diagnostic/therapeutic approaches for neurological diseases.

## Data Availability Statement

Publicly available datasets were analyzed in this study. This data can be found using the following accession numbers: PRJEB27306, SRP093968, PRJEB15418, PRJEB15420, PRJEB4927, PRJEB14674, and CRA000653.

## Author Contributions

HK and CB conceptualized the work and designed the experiments. HK performed the computational analysis with the assistance from CB. HK, CB, and SM analyzed the results and prepared the manuscript. All authors reviewed the results and approved the final manuscript.

## Conflict of Interest

HK, CB, and SM were employed by the company Tata Consultancy Services.

## Footnotes

1 https://www.uniprot.org/

2 ftp://ftp.ncbi.nlm.nih.gov/genomes/refseq/bacteria/

3 https://www.hmpdacc.org/hmp/HMRGD/

4 https://www.uniprot.org/

5 ftp://ftp.ncbi.nlm.nih.gov/genomes/refseq/bacteria/

6 https://www.hmpdacc.org/hmp/HMRGD/

7 https://cran.r-project.org/web/packages/gplots

8 https://www.uniprot.org/

9 genevenn.sourceforge.net/

10 https://cran.r-project.org/web/packages/gplots

11 https://plot.ly/python

12 ftp://ftp.ncbi.nlm.nih.gov/genomes/refseq/bacteria/

13 https://www.hmpdacc.org/hmp/HMRGD/
