# Supplementary material for: Tryptophan Metabolism by Gut Microbiome and Gut-Brain-Axis: An in silico Analysis
Source: Front Neurosci. 2019 Dec 18;13:1365. doi: 10.3389/fnins.2019.01365 (PMC6930238; doi:10.3389/fnins.2019.01365)
Supplement: Supplementary file 2 [file Table_2.pdf]

Supplementary Table S2: Details of the 16S rRNA gut microbiome datasets analyzed in the present study

Study	No. of samples		Site	Geography	Reference
Autism	Control	19	Duodenal Biopsy	US	(Kushak et al., 2017)
	Disease	21			
	Control	40	Stool	Italy	(Strati et al., 2017)
	Disease	40			
	Control	24	Stool	India	(Pulikkan et al., 2018)
	Disease	30			
Parkinson	Control	72	Stool	Finland	(Scheperjans et al., 2015)
	Disease	72			
	Control	130	Stool	US	(Hill-Burns et al., 2017)
	Disease	197			
Schizophrenia	Control	53	Stool	China	(Shen et al., 2018)
	Disease	64			

References

Hill-Burns, E. M., Debelius, J. W., Morton, J. T., Wissemann, W. T., Lewis, M. R., Wallen, Z. D., et al. (2017). Parkinson's Disease and PD Medications Have Distinct Signatures of the Gut Microbiome. *Mov. Disord. Off. J. Mov. Disord. Soc.* 32, 739–749. doi:10.1002/mds.26942.

Kushak, R. I., Winter, H. S., Buie, T. M., Cox, S. B., Phillips, C. D., and Ward, N. L. (2017). Analysis of the Duodenal Microbiome in Autistic Individuals: Association With Carbohydrate Digestion. *J. Pediatr. Gastroenterol. Nutr.* 64, e110–e116. doi:10.1097/MPG.0000000000001458.

Pulikkan, J., Maji, A., Dhakan, D. B., Saxena, R., Mohan, B., Anto, M. M., et al. (2018). Gut Microbial Dysbiosis in Indian Children with Autism Spectrum Disorders. *Microb. Ecol.* 76, 1102–1114. doi:10.1007/s00248-018-1176-2.

Scheperjans, F., Aho, V., Pereira, P. A. B., Koskinen, K., Paulin, L., Pekkonen, E., et al. (2015). Gut microbiota are related to Parkinson's disease and clinical phenotype. *Mov. Disord. Off. J. Mov. Disord. Soc.* 30, 350–358. doi:10.1002/mds.26069.

Shen, Y., Xu, J., Li, Z., Huang, Y., Yuan, Y., Wang, J., et al. (2018). Analysis of gut microbiota diversity and auxiliary diagnosis as a biomarker in patients with schizophrenia: A cross-sectional study. *Schizophr. Res.* doi:10.1016/j.schres.2018.01.002.

Strati, F., Cavalieri, D., Albanese, D., De Felice, C., Donati, C., Hayek, J., et al. (2017). New evidences on the altered gut microbiota in autism spectrum disorders. *Microbiome* 5, 24. doi:10.1186/s40168-017-0242-1.
